# MoO_3_ with the Synergistic Effect of Sulfur Doping and Oxygen Vacancies: The Influence of S Doping on the Structure, Morphology, and Optoelectronic Properties

**DOI:** 10.3390/nano14141189

**Published:** 2024-07-12

**Authors:** Jian Yu, Zhaokang Zheng, Aiwu Wang, Muhammad Humayun, Yasser A. Attia

**Affiliations:** 1 Shenzhen Key Laboratory of Ultraintense Laser and Advanced Material Technology, Center for Advanced Material Diagnostic Technology, College of Engineering Physics, Shenzhen Technology University, Shenzhen 518118, China; yujian@sztu.edu.cn (J.Y.); 2210412012@stumail.sztu.edu.cn (Z.Z.); 2 Energy, Water and Environment Lab, College of Humanities Sciences, Prince Sultan University, Riyadh 11586, Saudi Arabia; mhumayun@psu.edu.sa; 3 National Institute of Laser Enhanced Sciences, Cairo University, Giza 12613, Egypt; yassernile@niles.edu.eg

**Keywords:** sulfur doping, oxygen vacancy, Mott–Schottky, charge transfer

## Abstract

Molybdenum trioxide (MoO_3_) is an attractive semiconductor. Thus, bandgap engineering toward photoelectronic applications is appealing yet not well studied. Here, we report the incorporation of sulfur atoms into MoO_3_, using sulfur powder as a source of sulfur, via a self-developed hydrothermal synthesis approach. The formation of Mo-S bonds in the MoO_3_ material with the synergistic effect of sulfur doping and oxygen vacancies (designated as S-MoO_3−x_) is confirmed using Fourier-transform infrared (FTIR) spectroscopy, X-ray photoelectron spectroscopy (XPS), and electron paramagnetic resonance (EPR). The bandgap is tuned from 2.68 eV to 2.57 eV upon sulfur doping, as confirmed by UV-VIS DRS spectra. Some MoS_2_ phase is identified with sulfur doping by referring to the photoluminescence (PL) spectra and electrochemical impedance spectroscopy (EIS), allowing significantly improved charge carrier separation and electron transfer efficiency. Therefore, the as-prepared S-MoO_3−x_ delivers a sensitive photocurrent response and splendid cycling stability. This study on the synergistic effect of sulfur doping and oxygen vacancies provides key insights into the impact of doping strategies on MoO_3_ performance, paving new pathways for its optimization and development in relevant fields.

## 1. Introduction

Molybdenum trioxide (MoO_3_), as a representative of transition metal oxides, exhibits tremendous potential in both scientific research and practical applications. Its low cost, low toxicity, and excellent catalytic and optical properties make it an important material in various fields, such as hydrogen evolution reactions, lithium-ion batteries, optoelectronic devices, energy storage units, and catalysis [1,2,3,4,5]. Particularly at the nanoscale, where the physical dimensions of MoO_3_ are confined to zero-dimensional (0D) space or less than 10 nanometers, the quantum confinement effects and edge effects endow it with unique optical properties and easy functionalization, thereby greatly broadening the application prospects of MoO_3_ [6,7,8].

Doping is the most commonly used method for imparting new properties on quantum dots and nanomaterials [9]. Doping technology plays a crucial role in enhancing and expanding the performance of MoO_3_ materials. The electronic structure and surface properties of MoO_3_ can be significantly improved through doping which, as a result, optimizes its performance in various applications. For example, by introducing different metallic or non-metallic elements, the bandgap of MoO_3_ can be effectively tuned, thereby enhancing its gas sensitivity, photocatalytic efficiency, and electrochemical activity [10,11,12,13]. Moreover, doping can also improve the optical properties of MoO_3_, thereby enhancing its absorption in the visible light range and expanding its applications in optoelectronics and photonic devices [14,15,16]. The doping strategy not only alters the chemical composition of MoO_3_ but also introduces structural defects and surface modifications that can further improve the physical and chemical properties of a material. For instance, the introduction of oxygen vacancies can enhance the electron transport capability and catalytic activity of MoO_3_, which is crucial for battery electrode materials and catalysis [17,18,19,20]. It is worth mentioning that sulfur doping has been proven to narrow the bandgap of semiconductor materials and broaden their absorption range in the visible light region. Doping semiconductors with sulfur (S) can significantly improve their sensitivity to visible light [21].

Inspired by doping technology, this study reports the successful synthesis of novel MoO_3_ with the synergistic effect of sulfur doping and oxygen vacancies (designated as S-MoO_3−x_) through a simple solution process. Different characterization techniques, including TEM, XRD, FTIR, XPS, and UV-Vis DRS, were employed. Under relatively mild reaction conditions, a transformation from the molybdenum oxide phase to the molybdenum sulfide phase, involving the formation of Mo-S bonds, was observed. This process involved the substitution of oxygen atoms with sulfur atoms, resulting in significant changes in the material’s structure. Compared with bare α-MoO_3_ and MoO_3_ with oxygen vacancies introduced (denoted as MoO_3−x_), S-MoO_3−x_ exhibited superior optoelectronic properties. This study offers valuable insights into the effect of S doping on the performance of MoO_3_, presenting new opportunities for its further optimization and development in the fields of optoelectronics and catalysis.

## 2. Experimental Procedures

MoO_3_ powder with a purity of 99%, sulfur powder with a purity of 99%, and N-methyl-2-pyrrolidone (NMP) with a purity of 98% were acquired from Aladdin, a supplier based in Shanghai, China. Deionized water (MW 18) was utilized in all experiments, and all reagents were employed in their original forms without any additional purification.

### 2.1. Synthesis of S-MoO_3−x_ and MoO_3−x_

First, 0.12 g of MoO_3_ powder and 0.06 g of sulfur powder were added to a mix solvent containing 40 mL of deionized water and NMP at a ratio of 1:1. The mixture was then subjected to ultrasonication for 10 min before being transferred to a polytetrafluoroethylene-lined magnetic stirring reactor. The mixture was heated at 120 °C for 24 h. Subsequently, the sample was subjected to centrifugation at 12,000 rpm for 15 min in order to obtain the supernatant. The supernatant was then transferred to a freeze dryer and lyophilized for 24 h. The resulting blue-black powder obtained after lyophilization is referred to as S-MoO_3−x_. A similar method was used for the synthesis of MoO_3−x_, except for the addition of sulfur powder.

### 2.2. Characterization

The S-MoO_3−x_ and MoO_3−x_ solutions were placed on carbon-coated copper grids, dried under a vacuum, and observed using a transmission electron microscope (TEM) (JEOL, JEM-2100) operated at an accelerating voltage of 200 kV. The nanoparticle’s size was determined using dynamic light scattering (DLS) (i.e., the ZEN3600 instrument of Malvern Technologies in Shanghai, China). The X-ray diffraction patterns of the S-MoO_3−x_, MoO_3−x_, and α-MoO_3_ powder samples were measured in the range of 5–80° using the XRD technique (Japan Science Corporation, Tokyo, Japan, SmartLab XRD) with a Cu Kα radiation source (λ = 1.5406 Å). The vibrations of the chemical bonds were detected using the FTIR spectroscopic technique (Nicolet iS50 instrument; Thermo Fisher Scientific, Waltham, MA, USA). XPS analysis was performed using an X-ray photoelectron spectrometer (XPS) (XPS Escalab Xi+; Thermo Fisher Scientific (China) Co., Ltd., Shanghai, China). EPR analysis was performed with a Bruker (Shanghai, China) EMXplus-6/1 instrument to confirm the oxygen vacancies in the samples. A CHI-760E instrument from Shanghai CHENHUA (Shanghai, China) was used to measure the photocurrent response, impedance curves, and Mott–Schottky curves. During measurement, the Pt foil and Ag/AgCl (saturated KCl solution) served as the counter and reference electrodes, respectively. UV-visible diffuse reflectance spectroscopy (UV-vis DRS) analysis of the MoO_3−x_ and S-MoO_3−x_ samples was carried out using a UV-visible near-infrared spectrophotometer (Lambda 1050+; PerkinElmer Enterprise Management (Shanghai) Co., Ltd., Shanghai, China) equipped with an integrating sphere.

### 2.3. Current–Voltage Curve Testing

First, we coated the interdigital electrodes with the MoO_3−x_ and S-MoO_3−x_ solutions. Then, we placed them on a heating platform to dry the solutions. We repeated the coating-drying steps 3–4 times to obtain the samples for testing. We then placed the samples on a semiconductor probe station (YB600-A; YINGBO SCIENCE & INSTRUMENT, Shanghai, China) for testing, ensuring the entire testing process was conducted in a photoelectric dark box.

## 3. Results and Discussion

**Figure 1:**
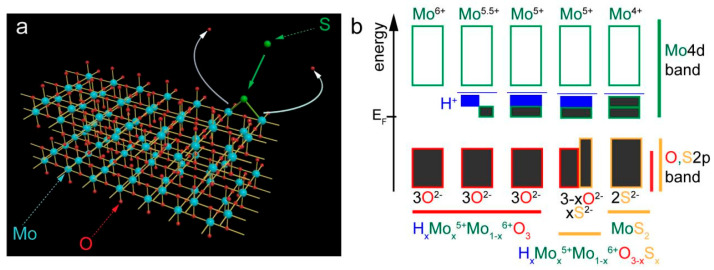
(**a**) Schematic of the S–doped MoO_3−x_. (**b**) Changes in electronic structure due to hydrogen intercalation and S doping. Bandgap states formed by hydrogen intercalation (blue) and “valence band-like Mo^5+^ state” (in the green box, black means occupied, and white means unoccupied), as indicated by the widths of the electronic states.

Typically, MoO_3_ exists in the form of a layered structure, where Mo atoms are surrounded by six O atoms forming octahedral structural units [22]. However, in S-MoO_3−x_, the structure of the material underwent changes due to the substitution of S atoms. This substitution led to the formation of Mo–S bonds (as depicted in Figure 1a), which resulted in the transition of oxide to a sulfide state. The emergence of Mo–S bonds implies the replacement of O atom positions with S atoms, which exhibit different electronic and chemical features. Meanwhile, the Mo–O bonds in MoO_3_ are primarily ionic, and the bonds between Mo and S display more metallic sulfide characteristics [22,23]. This transition not only induces significant changes in the chemical properties but also triggers adjustments in various aspects such as the electrical and optical properties, as elaborated subsequently.

Figure 1b depicts the process of hydrogen intercalation (essentially the appearance of O vacancies) and S doping-induced changes in the electronic band structure of MoO_3_. This illustrates a gradual transition from MoO_3_ to MoS_2_. In the undoped MoO_3_, as depicted on the far left, Mo exists in its highest oxidation state (Mo^6+^), corresponding to a fully occupied oxygen 2p band, which displays typical wide bandgap insulator characteristics. In this scenario, due to the lack of free charge carriers in the conduction band, the material exhibited low electrical conductivity. In the case of the hydrogen intercalation bandgap states (H_x_Mo_x_^5+^Mo_1−x_^6+^O_3_), when hydrogen atoms were inserted into the material, some Mo^6+^ ions were reduced to Mo^5+^ with the appearance of O vacancies, as reflected by the changes in the partially filled Mo 4d band (see Figure 1b). Electron filling resulted in a reduced bandgap and increased conductivity of the material, a key feature in electrochemical storage and chemical sensors [17,24]. In the case of the intermediate state of S doping (H_x_Mo_x_^5+^Mo_1−x_^6+^O_3−x_S_x_), with the introduction of S, the original MoO_3_ structure was partially replaced by S, resulting in a composite material containing a small amount of the MoS_2_ phase. During this process, the oxygen 2p band is affected, as it is partially replaced by the sulfur 2p band, leading to adjustments in the band structure which may influence the optical and electrical properties of the material [25,26]. In the case of the MoS_2_ state, on the far right of this series, complete S doping resulted in the formation of MoS_2_. In MoS_2_, the oxidation state of Mo further reduced to Mo^4+^, with each Mo atom coordinated with two S atoms (S^2−^), forming a semiconductor with a smaller bandgap. Due to there being more electrons in the conduction band, this structural change significantly enhanced the conductivity of the material. In summary, Figure 1b demonstrates how hydrogen intercalation and S doping regulate the valence states and electronic band structure of MoO_3_, enabling precise control over its electronic, optical, and chemical properties. Such control is crucial for developing new functional materials and optimizing the performance of existing materials.

**Figure 2:**
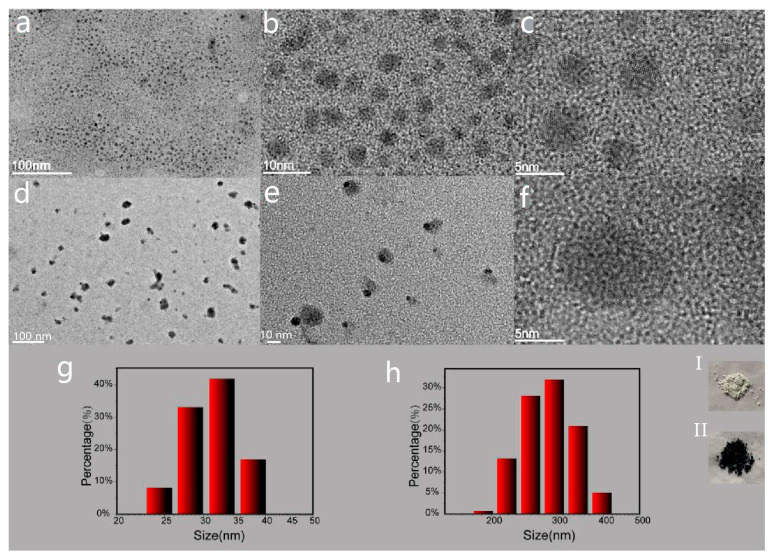
(**a**–**c**) TEM images of MoO_3−x_. (**d**–**f**) TEM images of S-MoO_3−x_. (**g**) DLS spectrum of MoO_3−x_. (**h**) DLS spectrum of S-MoO_3−x_. (**h**, **I**) Physical image of α-MoO_3_ powder. (**h**, **II**) Physical image of S-MoO_3−x_ powder.

The TEM images of MoO_3−x_ are shown in Figure 2a–c, and the TEM images of S-MoO_3−x_ are provided in Figure 2d–f. It can be observed that MoO_3−x_ exhibited a more uniform dispersion, while S-MoO_3−x_ tended to aggregate more easily. The average size of the MoO_3−x_ particles was roughly a few nanometers, whereas for S-MoO_3−x_, it was approximately tens of nanometers, and lattice fringes can be seen in the high-resolution TEM images of both materials (Figure 2c,f). However, the DLS spectra (Figure 2g,h) show that the average size of MoO_3−x_ was approximately 30 nanometers, while that of S-MoO_3−x_ was about 300 nanometers, contradicting the TEM results. Nevertheless, the increase in statistical size can be attributed to the aggregation of small particles, which also serves as evidence for the easier aggregation of S-MoO_3−x_. The color change of the α-MoO_3_ powder from gray-white to blue-black, corresponding to the S-MoO_3−x_ powder, is the most direct evidence of the change in the band structure of the material.

**Figure 3:**
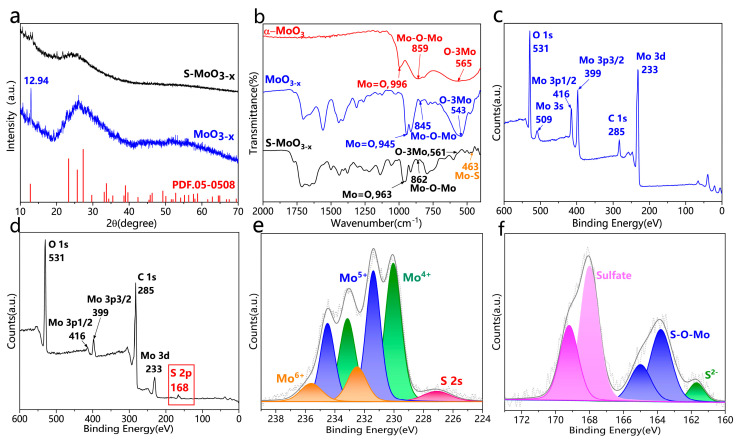
(**a**) XRD spectra of MoO_3−x_ and S-MoO_3−x_. (**b**) Fourier transform infrared spectra of α-MoO_3_, MoO_3−x_, and S-MoO_3−x_. (**c**) XPS survey spectrum of MoO_3−x_. (**d**) XPS survey spectrum of S-MoO_3−x_. (**e**) High–resolution XPS spectrum of the Mo 3d region in S-MoO_3−x_. (**f**) High-resolution XPS spectrum of the S 2p region in S-MoO_3−x_.

The crystalline phase of the MoO_3−x_ nanostructures is depicted in Figure 3a. In the range from 20° to 40°, the broadening of sharp diffraction peaks can be attributed to a large number of severe lattice defects caused by oxygen vacancies. The sharp diffraction peak at 12.94° can be attributed to the α-MoO_3_ (020) crystal plane (JCPDF No. 05-0508) [27], suggesting that MoO_3−x_ nanostructures preferentially form along the [0k0] direction. To further investigate the structural characteristics of S-MoO_3−x_, we also obtained the crystalline phase of S-MoO_3−x_ through XRD analysis. By comparing this with the crystalline phase of the MoO_3−x_ nanostructures, it is evident that S-MoO_3−x_ also exhibited an amorphous structure. The disappearance of the diffraction peak at 12.94° indicates the absence of the (020) crystal plane in S-MoO_3−x_, which could be attributed to the introduction of more O vacancy defects during S doping. The FTIR spectra in the range of 400–2000 cm^−1^ (Figure 3b) confirms the successful doping of S in the S-MoO_3−x_ (i.e., the formation of Mo-S bonds). In the α-MoO_3_ sample, three peaks were observed; the peak at 996 cm^−1^ corresponds to the stretching vibration of terminal Mo=O, indicating its orthorhombic layered MoO_3_ structure, the peak at 859 cm^−1^ represents the symmetric vibration of Mo–O−Mo bonds, and the peak at 565 cm^−1^ was due to the bending vibration of O atoms bonded to three metal atoms (O-3Mo) [28,29]. Both the MoO_3−x_ and S-MoO_3−x_ samples exhibited peaks at 945, 845, and 543 cm^−1^ as well as 963, 862, and 561 cm^−1^, attributed to the vibration modes of the Mo=O, Mo–O−Mo, and O−3Mo bonds, respectively [11,30]. In the S-MoO_3−x_ sample, the peak at 463 cm^−1^ represents the vibration modes of the Mo−S bonds [31,32], providing evidence of the formation of the MoS_2_ phase as facilitated by the S atoms through O vacancies.

**Figure 4:**
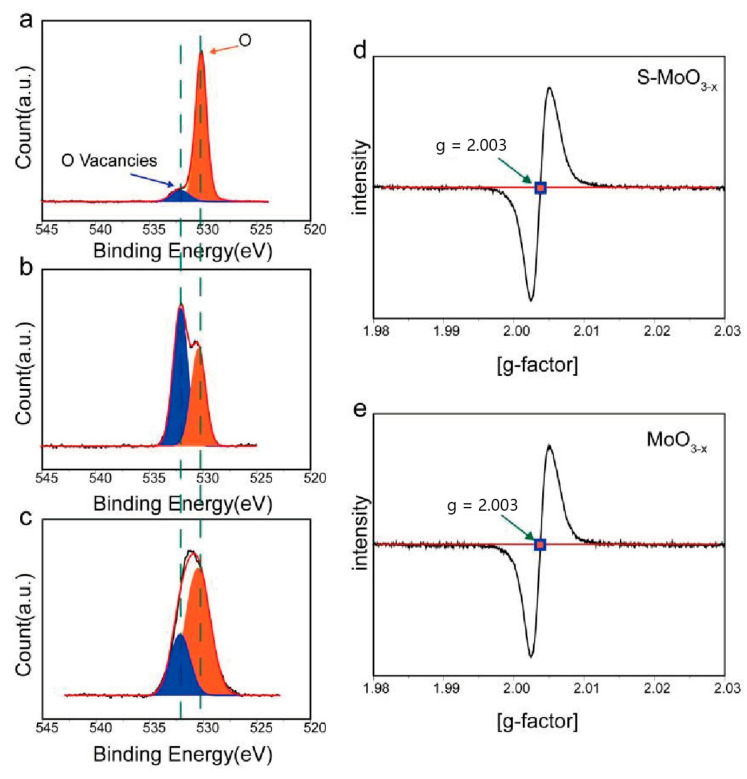
(**a**) High–resolution XPS spectrum of the O 1s region for α-MoO_3_. (**b**) High–resolution XPS spectrum of the O 1s region for S-MoO_3−x_. (**c**) High-resolution XPS spectrum of the O 1s region for MoO_3−x_. (**d**) EPR spectrum of S-MoO_3−x_. (**e**) EPR spectrum of MoO_3−x_.

XPS analysis was conducted to further investigate the chemical composition and elemental chemical states of the samples. The XPS survey spectra of MoO_3−x_ (Figure 3c) and S-MoO_3−x_ (Figure 3d) revealed that the main difference between both samples was the appearance of spin-orbit splitting peaks for S2p at approximately 168 eV in the S-MoO_3−x_ sample, evidencing the successful doping of sulfur. Figure 3e shows the high-resolution XPS spectrum of the Mo 3d region, which revealed two orbital splitting peaks at a lower binding energy (i.e., 3d_5/2_) and a higher binding energy (i.e., 3d_3/2_). The peaks at binding energies (BEs) of 230 (Mo3d_5/2_^4+^) and 233.1 eV (Mo3d_3/2_^4+^) can be attributed to the Mo−S bond [33]. The peaks at BEs of 231.4 (Mo3d_5/2_^5+^) and 234.5 eV (Mo3d_5/2_^5+^) can be attributed to MoO_3−x_. The peaks at BEs of 232.5 (Mo3d_5/2_^6+^) and 235.6 eV (Mo3d_5/2_^6+^) are ascribed to MoO_3_ [34]. In addition, a weak S 2s peak was detected at 227 eV, confirming the existence of MoS_2_ [33,34]. Furthermore, the S 2p core-level XPS spectrum of the S-MoO_3−x_ sample is revealed in Figure 3f. A broad peak was deconvoluted into four peaks centered at 161.6, 162.8, 163.7, and 164.9 eV, corresponding to the S 2p_3/2_ (S^2−^), S 2p_1/2_ (S^2−^), S 2p_3/2_ (S–O–Mo), and S 2p_1/2_ (S–O–Mo) levels, respectively. This further indicates the existence of terminal S^2−^ (corresponding to Mo–S) and S–O–Mo bonding in S-MoO_3−x_ [35]. The peaks at 168 and 169.2 eV belong to the sulfate [36]. To further investigate the existence of sulfate bonds in S-MoO_3−x_, we performed high-resolution XPS spectroscopy analysis of the O 1s region of the α-MoO_3_, MoO_3−x_, and S-MoO_3−x_ samples. As shown in Figure 4a–c, for all three samples, two peaks centered at 530.2 and 532.3 eV were observed, which correspond to the lattice oxygen and O vacancies in MoO_3_, respectively [37,38]. Among them, α-MoO_3_ exhibited the lowest content of O vacancies, while S-MoO_3−x_ showed the highest one. EPR analysis of the MoO_3−x_ and S-MoO_3−x_ samples was further conducted, and the results are depicted in Figure 4d,e. Peaks with a g value of 2.003 were observed in both the S-MoO_3−x_ and MoO_3−x_ samples, which further provide evidence of the existence of O vacancies in both samples [39,40]. The analyses of XPS and EPR indicate that only lattice oxygen and O vacancies existed in S-MoO_3−x_, and there was no formation of SO_3_^2^ or SO4^2^ involving O or S. Therefore, the sulfate bond was attributed to the S–O bonding between the S atoms at the edge of MoS_2_ formed by the interaction of doped S and the O atoms of MoO_3_. This S–O bond suggests a covalent interaction between MoO_3−x_ and MoS_2_. Such covalent bonds indicate a close contact between MoO_3_ and MoS_2_, which facilitates the transfer of electrons [41,42].

**Figure 5:**
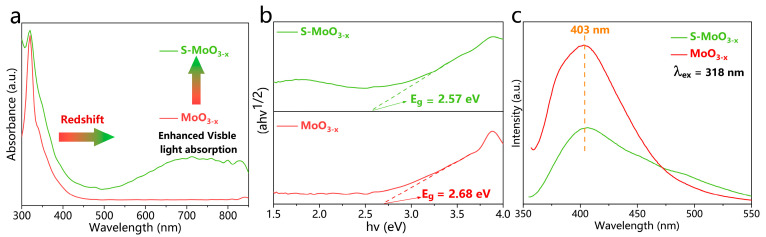
(**a**) UV–VIS DRS spectra of S-MoO_3−x_ and MoO_3−x_ samples. (**b**) Curves of (αhν) ^1/2^ versus hν for S-MoO_3−x_ and MoO_3−x_ samples. (**c**) Photoluminescence spectra of MoO_3−x_ and S-MoO_3−x_.

**Figure 6:**
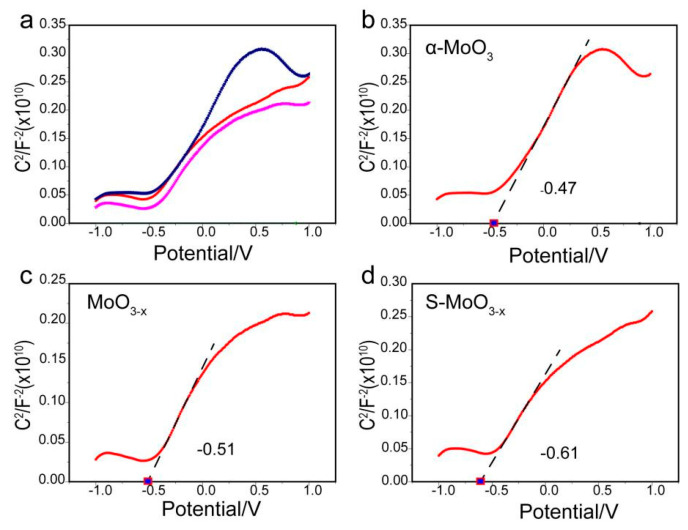
(**a**) Mott–Schottky plot of α-MoO_3_, MoO_3−x_, and S-MoO_3−x_ at fixed frequency of 1 kHz, (**b**) Mott–Schottky plot of α-MoO_3_, (**c**) Mott–Schottky plot of MoO_3−x_, and (**d**) Mott–Schottky plot of S-MoO_3−x_.

To investigate the influence of S doping on the optoelectronic properties of oxygen-deficient molybdenum oxide semiconductor materials, the S-MoO_3−x_ and MoO_3−x_ samples were prepared and subjected to diffuse reflectance spectroscopy (DRS), photoluminescence (PL) spectroscopy, and Mott–Schottky analysis. As shown in Figure 5a, MoO_3−x_ exhibited a cutoff absorption edge at approximately 430 nm, while the S-MoO_3−x_ sample demonstrated a redshift in the absorption edge compared with MoO_3−x_, indicating enhanced light-harvesting properties attributed to S doping. This leads to the formation of the MoS_2_ phase, which extends the light absorption capability of the material [43]. The corresponding main band structures of the MoO_3−x_ and S-MoO_3−x_ samples (Figure 5b) in the visible light region were calculated with the transformed Kubelka–Munk function (i.e., αhv = A(hv − Eg)^n/2^), where α is the absorption coefficient, hv is the photon energy, n is related to the type of semiconductor (the value of n is taken to be one for direct bandgap semiconductors and four for indirect bandgap semiconductors), Eg is the band gap, and A is a constant. Since MoO_3_ is essentially an indirect bandgap semiconductor, its n value was taken to be four [44]. In Figure 5b, it can be observed that the bandgaps of MoO_3−x_ and S-MoO_3−x_ were measured to be 2.68 and 2.57 eV, respectively, both of which were smaller than that of the bare α-MoO_3_ (Eg = 3 eV) [22]. To investigate the separation of the photo-generated electron-hole pairs in S-MoO_3−x_, PL analysis was performed. Figure 5c illustrates the PL spectra of MoO_3−x_ and S-MoO_3−x_, both of which exhibited peaks centered at 403 nm. Interestingly, the emission peak intensity of S-MoO_3−x_ was weaker than that of MoO_3−x_, confirming the rapid charge transfer between MoO_3_ and sulfide. This indicates a high separation efficiency of photo-generated electron-hole pairs in S-MoO_3−x_, resulting in effective suppression of carrier recombination [45,46] and being consistent with the XPS results. As depicted in Figure 6a, the Mott–Schottky plot reveals a significant influence of O vacancies and S doping on the flat band potential (E_FB_) of MoO_3_. The Mott–Schottky curves of α-MoO_3_, MoO_3−x_, and S-MoO_3−x_ all exhibit positive slopes, which indicates the typical n-type semiconductor behavior. With the appearance of O vacancies and successful doping of S, E_FB_ underwent a negative shift, suggesting an enhanced ease in the charge transfer process [47,48].

The E_FB_ were estimated to be −0.47, −0.51, and −0.61 V versus the Ag/AgCl for α-MoO_3_, MoO_3−x_, and S-MoO_3−x_, respectively. The conduction band potentials (E_CB_s) of the n-type semiconductors were approximately 0.1 or 0.2 V lower than their flat band potentials [49]. Thus, the E_CB_s for MoO_3−x_ and S-MoO_3−x_ were estimated to be about −0.71 and −0.81 V versus the Ag/AgCl, corresponding to −0.51 and −0.61 V versus the normal hydrogen electrode (NHE) (E_NHE_ = E_Ag/AgCl_ + 0.197 V), respectively [50]. Combined with the bandgap values obtained through UV-VIS DRS analysis (Figure 5b), the valence band potentials (E_VB_s) of MoO_3−x_ and S-MoO_3−x_ were calculated using the equation E_VB_ = E_CB_ + E_g_ [51]. More clearly, the E_CB_ and E_VB_ positions of MoO_3−x_ and S-MoO_3−x_ are summarized in Table 1.

**Figure 7:**
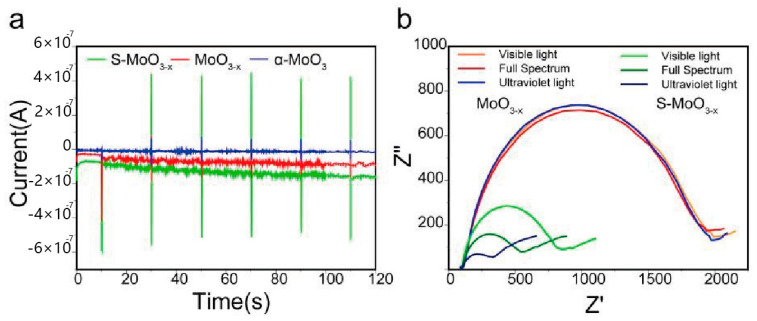
(**a**) Transient photocurrent responses of α-MoO_3_, MoO_3−x_, and S-MoO_3−x_ under ultraviolet light illumination. (**b**) Electrochemical impedance spectroscopy (EIS) Nyquist plots of MoO_3−x_ and S-MoO_3−x_ under visible light, full spectrum, and ultraviolet light illumination.

**Figure 8:**
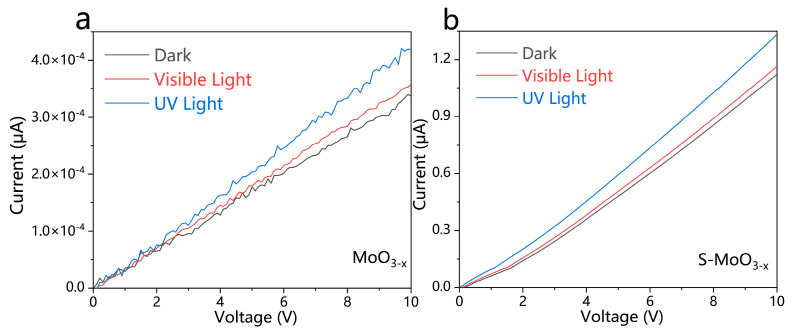
(**a**,**b**) Current–voltage curves of MoO_3−x_ and S-MoO_3−x_ under dark, visible light, and ultraviolet light illumination.

The transient photocurrent results, illustrated in Figure 7a, indicate that S-MoO_3−x_ exhibited a sensitive photocurrent response under visible light intermittent irradiation over several switching cycles. Among these three samples, S-MoO_3−x_ demonstrated the highest photocurrent intensity under visible light irradiation, reflecting its superior ultraviolet light response and more effective charge separation upon light excitation [52,53]. This is consistent with the PL results (Figure 5c). Additionally, with the increase in switching cycles, the photocurrent intensity of the S-MoO_3−x_ sample showed no significant attenuation, implying no electron-hole recombination during cycling and confirming good cycling stability [50]. In contrast, the photocurrent intensity of the MoO_3−x_ sample gradually decreased with the increase in switching cycles, possibly due to the formation of defects (O vacancies) induced by light irradiation [54,55]. Furthermore, EIS Nyquist plots were also employed to investigate the advantage of S-MoO_3−x_ over MoO_3−x_ in the process of photo-generated electron-hole pair transfer. As shown in Figure 7b, we observed smaller semicircles for S-MoO_3−x_ compared with MoO_3−x_ under visible light, full spectrum, and ultraviolet light irradiation. This further demonstrates the effective charge separation and faster charge transfer in S-MoO_3−x_ compared with MoO_3−x_ [56,57]. Under visible light, full spectrum, and ultraviolet light irradiation, S-MoO_3−x_ exhibited semicircles with different diameters, indicating that S-MoO_3−x_ shows different charge transfer resistances (R_ct_) under different conditions. The largest semicircle diameter was observed under visible light irradiation, with a fitted R_ct_ of 771 Ω. The semicircle diameter was intermediate under full spectrum light irradiation, with a fitted R_ct_ of 511 Ω. The smallest semicircle was observed under ultraviolet light irradiation, with an R_ct_ of 296 Ω. These results demonstrate that S-MoO_3−x_ had the lowest charge transfer resistance under ultraviolet light irradiation [58]. The charge transfer resistance of MoO_3−x_ showed almost no change in these three cases, and after fitting, R_ct_ = 1941 Ω. The current–voltage curve (Figure 8) also shows that the resistance of MoO_3−x_ was much greater than that of S-MoO_3−x_. However, the photo-response values of both materials exhibited the same phenomenon; the current was highest under UV light followed by visible light, and it was lowest in darkness. For S-MoO_3−x_, both the reduction in resistance under light exposure and the generation of photocurrent contributed to the increase in current. In contrast, for MoO_3−x_, we believe that the increase in current was solely due to the generation of photocurrent. This result is also consistent with the analysis in Figure 7.

## 4. Conclusions

In summary, we successfully fabricated S-MoO_3−x_ nanostructures via the hydrothermal process and systematically investigated the effect of S doping on the morphology and optoelectronic properties of MoO_3_. The introduction of S atoms substituted some oxygen atoms and generated MoS_2_ with the S atoms bonded to oxygen atoms in the MoO_3_, resulting in S–O bonds. The MoS_2_ phase formed by S doping exhibited dual functionality, acting as a sensitizing unit to induce visible light response and enhancing the rate of the charge carrier’s separation. Therefore, due to the enhanced visible light absorption and rapid charge carrier’s separation, S-MoO_3−x_ holds promise as a potential photocatalytic material. This work presents a simple and efficient strategy for modifying MoO_3_ with S doping which significantly improves the optoelectronic performance of MoO_3_ compared with the sole oxygen vacancies introduced into MoO_3_.

## 5. Patents

This work is related to Chinese Patent Application Number 2022110784700.

## Figures and Tables

**Table 1:** Calculated E_CB_ and E_VB_ values for MoO_3−x_ and S-MoO_3−x_.

Semiconductor	E_g_ (eV)	E_CB_ (eV)	E_VB_ (eV)
MoO_3−x_	2.68	−0.51	2.17
S-MoO_3−x_	2.57	−0.61	1.96

## Data Availability

Dataset available on request from the authors.
